# Methyl 1-[(*Z*)-2-(benzyloxycarbonyl)hydrazin-1-ylidene]-5-chloro-2-hydroxy­indane-2-carboxylate

**DOI:** 10.1107/S1600536814007429

**Published:** 2014-04-09

**Authors:** Kun Dong, Yifeng Wang

**Affiliations:** a Catalytic Hydrogenation Research Center, Zhejiang University of Technology, Hangzhou, 310014, People’s Republic of China

## Abstract

The title compound, C_19_H_17_ClN_2_O_5_, is an important inter­mediate for the synthesis of the pesticide Indoxacarb [systematic name: (*S*)-methyl 7-chloro-2-{[(meth­oxy­carbon­yl)[4-(tri­fluoro­meth­oxy)phen­yl]amino]­carbon­yl}-2*H*,3*H*,4a*H*,5*H*-indeno­[1,2-*e*][1,3,4]oxadiazine-4a-carboxyl­ate] The C=N double bond has a *Z* conformation. An intra­molecular N—H⋯O hydrogen bond occurs. In the crystal structure, O—H⋯O hydrogen bonds result in the formation of 12-membered rings lying about inversion centers with *R* ^4^_4_(12) motifs.

## Related literature   

For the synthesis of the title compound, see: Annis *et al.* (1991 ▶); Annis (1995 ▶). For graph-set notation, see: Bernstein *et al.* (1995 ▶).
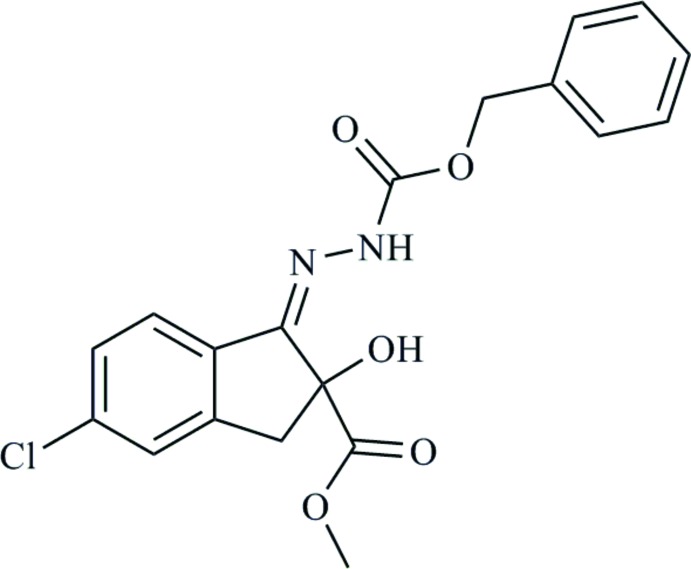


## Experimental   

### 

#### Crystal data  

C_19_H_17_ClN_2_O_5_
*M* *_r_* = 388.79
Triclinic, 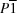

*a* = 8.362 (3) Å
*b* = 10.627 (3) Å
*c* = 11.470 (4) Å
α = 108.575 (6)°
β = 99.377 (7)°
γ = 100.886 (6)°
*V* = 921.1 (5) Å^3^
*Z* = 2
Mo *K*α radiation
μ = 0.24 mm^−1^
*T* = 293 K
0.21 × 0.18 × 0.12 mm

#### Data collection  

Bruker SMART CCD area-detector diffractometer
Absorption correction: multi-scan (*SADABS*; Sheldrick, 2000 ▶) *T*_min_ = 0.587, *T*_max_ = 0.746
5494 measured reflections
3569 independent reflections
2965 reflections with *I* > 2σ(*I*)
*R*_int_ = 0.030

#### Refinement  

*R*[*F* ^2^ > 2σ(*F* ^2^)] = 0.053
*wR*(*F* ^2^) = 0.162
*S* = 1.03
3569 reflections
251 parameters
H atoms treated by a mixture of independent and constrained refinement
Δρ_max_ = 0.35 e Å^−3^
Δρ_min_ = −0.31 e Å^−3^

### 

Data collection: *SMART* (Bruker, 2013 ▶); cell refinement: *SAINT* (Bruker, 2013 ▶); data reduction: *SAINT*; program(s) used to solve structure: *SHELXTL* (Sheldrick, 2008 ▶); program(s) used to refine structure: *SHELXL2013* (Sheldrick, 2008 ▶); molecular graphics: *SHELXTL*; software used to prepare material for publication: *SHELXTL*.

## Supplementary Material

Crystal structure: contains datablock(s) I. DOI: 10.1107/S1600536814007429/pk2520sup1.cif

Structure factors: contains datablock(s) I. DOI: 10.1107/S1600536814007429/pk2520Isup2.hkl

Supporting information file. DOI: 10.1107/S1600536814007429/pk2520Isup3.cml

CCDC reference: 995214

Additional supporting information:  crystallographic information; 3D view; checkCIF report

## Figures and Tables

**Table 1:** Hydrogen-bond geometry (Å, °)

*D*—H⋯*A*	*D*—H	H⋯*A*	*D*⋯*A*	*D*—H⋯*A*
O1—H1⋯O2^i^	0.82	2.13	2.890 (2)	154
N2—H2*A*⋯O1	0.86 (3)	2.37 (2)	2.891 (2)	119.4 (18)

Symmetry code: (i) 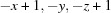.

**Fig. 1.**
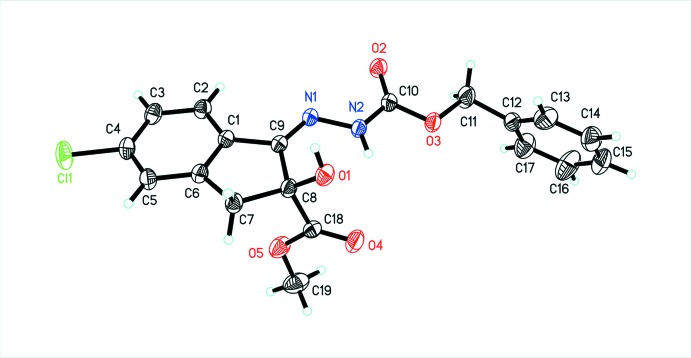
The structure of the title compound. Displacement ellipsoids are drawn at the 50% probability level.

**Fig. 2.**
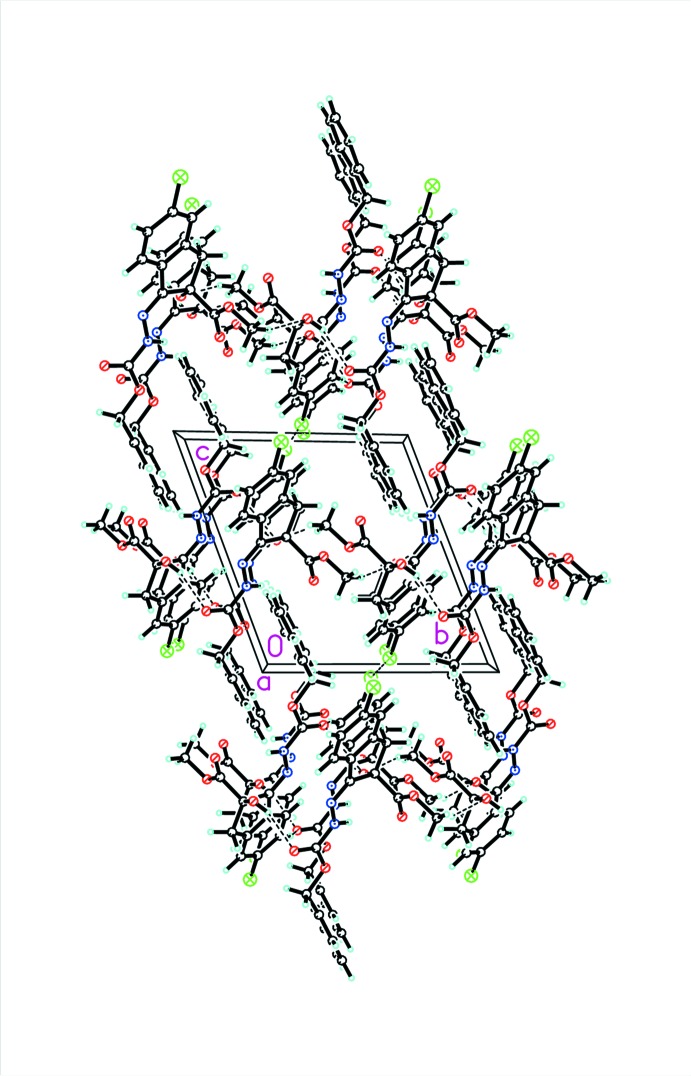
The crystal packing of the title compound.

## supplementary crystallographic information

### 1. Comment

The title compound, which was readily synthesized from the condensation of methyl 5-chloro-1,3-dihydro-2-hydroxy-1-oxo-2*H*-inden-2-carboxylate and phenylmethyl hydrazinecarboxylate, acts as an intermediate for the synthesis of Indoxacarb. In this article, the crystal structure of the title compound is presented (Fig. 1). The C=N double bond has a *Z* configuration. The N2—N1—C9—C8 torsion angle is 4.33 (3)°. The crystal structure is stabilized by intermolecular O1—H1···O2 and intramolecular N2—H2A···O1 hydrogen bonds, resulting in the formation of twelve-membered ring lying about an inversion center and representing *R*^4^_4_(12) motif. In the crystal, there are weak intermolecular Cl···Cl interactions, with a distance of 3.379 (2) Å. The hydroxyl O atom lies 0.584 (2) Å from the mean plane of the phenyl ring C1···C6. The benzyl C atom lies 0.129 (2) Å from the mean plane of the phenyl ring C12···C17. The dihedral angle of the planes of two phenyl rings is 73.33 (3)°.

### 2. Experimental

In a reaction flask, methyl 5-chloro-1,3-dihydro-2-hydroxy-1-oxo-2*H*-inden-2- carboxylate (11.20 g, 0.05 mol), phenylmethyl hydrazinecarboxylate (9.13 g, 0.055 mol) and *p*-toluenesulfonic acid monohydrate (0.95 g, 0.005 mol) were added to toluene (55 ml). The mixture was stirred at 66°C for 8 h. After completion of the reaction (monitored by HPLC), the mixture was cooled to room temperature and filtered. The filter cake was washed with cold methanol (20 ml) and dried at 40°C under vacuum for 4 h, giving a white solid. Single crystals were obtained by slow evaporation of a methanol and dichloromethane solution.

### 3. Refinement

H atoms attached to C and O atoms were placed in calculated positions with O—H = 0.82 Å, C—H = 0.97 (methylene), 0.96 (methyl), 0.93 Å (aromatic H atoms), and *U*_iso_(H) = 1.2*U*_eq_ (C, O) in the riding model approximation. The H atom attached to N was located in a difference Fourier map and refined with a distance restraint of N—H = 0.86 (1) Å and *U*_iso_(H) = 1.5*U*_eq_ (N).

### Crystal data

**Table:**

C_19_H_17_ClN_2_O_5_	*Z* = 2
*M**_r_* = 388.79	*F*(000) = 404
Triclinic, *P*1	*D*_x_ = 1.402 Mg m^−^^3^
*a* = 8.362 (3) Å	Mo *K*α radiation, λ = 0.71073 Å
*b* = 10.627 (3) Å	Cell parameters from 2385 reflections
*c* = 11.470 (4) Å	θ = 2.6–28.1°
α = 108.575 (6)°	µ = 0.24 mm^−^^1^
β = 99.377 (7)°	*T* = 293 K
γ = 100.886 (6)°	Prismatic, colorless
*V* = 921.1 (5) Å^3^	0.21 × 0.18 × 0.12 mm

### Data collection

**Table:**

Bruker SMART CCD area-detector diffractometer	2965 reflections with *I* > 2σ(*I*)
φ and ω scans	*R*_int_ = 0.030
Absorption correction: multi-scan (*SADABS*; Sheldrick, 2000)	θ_max_ = 26.0°, θ_min_ = 1.9°
*T*_min_ = 0.587, *T*_max_ = 0.746	*h* = −9→10
5494 measured reflections	*k* = −13→11
3569 independent reflections	*l* = −12→14

### Refinement

**Table:**

Refinement on *F*^2^	Hydrogen site location: mixed
Least-squares matrix: full	H atoms treated by a mixture of independent and constrained refinement
*R*[*F*^2^ > 2σ(*F*^2^)] = 0.053	*w* = 1/[σ^2^(*F*_o_^2^) + (0.0957*P*)^2^ + 0.1511*P*] where *P* = (*F*_o_^2^ + 2*F*_c_^2^)/3
*wR*(*F*^2^) = 0.162	(Δ/σ)_max_ = 0.001
*S* = 1.03	Δρ_max_ = 0.35 e Å^−^^3^
3569 reflections	Δρ_min_ = −0.31 e Å^−^^3^
251 parameters	Extinction correction: *SHELXL*, Fc^*^=kFc[1+0.001xFc^2^λ^3^/sin(2θ)]^-1/4^
0 restraints	Extinction coefficient: 0.064 (8)

### Special details

**Table:**

Geometry. All e.s.d.'s (except the e.s.d. in the dihedral angle between two l.s. planes) are estimated using the full covariance matrix. The cell e.s.d.'s are taken into account individually in the estimation of e.s.d.'s in distances, angles and torsion angles; correlations between e.s.d.'s in cell parameters are only used when they are defined by crystal symmetry. An approximate (isotropic) treatment of cell e.s.d.'s is used for estimating e.s.d.'s involving l.s. planes.

### Fractional atomic coordinates and isotropic or equivalent isotropic displacement parameters (Å^2^)

**Table:**

	*x*	*y*	*z*	*U*_iso_*/*U*_eq_	
Cl1	1.28901 (7)	0.44224 (8)	0.95091 (6)	0.0735 (3)	
N1	0.63739 (19)	0.07412 (16)	0.44508 (14)	0.0374 (4)	
N2	0.5019 (2)	0.05154 (17)	0.34896 (14)	0.0402 (4)	
O1	0.41582 (16)	0.25433 (16)	0.54793 (13)	0.0463 (4)	
H1	0.4022	0.2091	0.5933	0.070*	
O2	0.52986 (18)	−0.16042 (15)	0.24204 (13)	0.0494 (4)	
O3	0.35071 (19)	−0.06312 (15)	0.15431 (13)	0.0510 (4)	
O4	0.4938 (2)	0.35295 (19)	0.37465 (16)	0.0689 (5)	
O5	0.76048 (19)	0.43718 (19)	0.48013 (16)	0.0614 (5)	
C1	0.8288 (2)	0.23034 (19)	0.63817 (17)	0.0361 (4)	
C2	0.9552 (2)	0.1639 (2)	0.65007 (18)	0.0433 (5)	
H2	0.9448	0.0765	0.5928	0.052*	
C3	1.0959 (2)	0.2294 (2)	0.74786 (19)	0.0479 (5)	
H3	1.1822	0.1870	0.7577	0.057*	
C4	1.1070 (2)	0.3591 (2)	0.83115 (19)	0.0475 (5)	
C5	0.9811 (2)	0.4255 (2)	0.82290 (18)	0.0464 (5)	
H5	0.9912	0.5124	0.8812	0.056*	
C6	0.8395 (2)	0.3583 (2)	0.72483 (17)	0.0395 (4)	
C7	0.6855 (2)	0.4073 (2)	0.69572 (18)	0.0448 (5)	
H7A	0.7159	0.5003	0.6964	0.054*	
H7B	0.6186	0.4049	0.7569	0.054*	
C8	0.5888 (2)	0.3050 (2)	0.56156 (17)	0.0389 (4)	
C9	0.6780 (2)	0.18852 (19)	0.53719 (16)	0.0354 (4)	
C10	0.4663 (2)	−0.0664 (2)	0.24796 (18)	0.0403 (4)	
C11	0.3026 (4)	−0.1873 (3)	0.0421 (2)	0.0729 (8)	
H11A	0.2559	−0.2656	0.0632	0.087*	
H11B	0.4004	−0.2028	0.0101	0.087*	
C12	0.1753 (3)	−0.1721 (2)	−0.05686 (19)	0.0504 (5)	
C13	0.0110 (4)	−0.1859 (3)	−0.0493 (2)	0.0678 (7)	
H13	−0.0200	−0.1974	0.0216	0.081*	
C14	−0.1088 (3)	−0.1830 (3)	−0.1462 (3)	0.0735 (8)	
H14	−0.2196	−0.1921	−0.1400	0.088*	
C15	−0.0656 (4)	−0.1668 (3)	−0.2496 (3)	0.0763 (8)	
H15	−0.1470	−0.1677	−0.3158	0.092*	
C16	0.0958 (4)	−0.1493 (4)	−0.2567 (3)	0.0977 (12)	
H16	0.1262	−0.1346	−0.3268	0.117*	
C17	0.2163 (3)	−0.1528 (3)	−0.1612 (3)	0.0761 (8)	
H17	0.3270	−0.1420	−0.1681	0.091*	
C18	0.6054 (2)	0.3681 (2)	0.46057 (19)	0.0431 (5)	
C19	0.7984 (4)	0.5012 (3)	0.3910 (3)	0.0746 (8)	
H19A	0.7713	0.4323	0.3077	0.112*	
H19B	0.9157	0.5471	0.4146	0.112*	
H19C	0.7335	0.5668	0.3914	0.112*	
H2A	0.450 (3)	0.114 (3)	0.351 (2)	0.056 (7)*	

### Atomic displacement parameters (Å^2^)

**Table:**

	*U*^11^	*U*^22^	*U*^33^	*U*^12^	*U*^13^	*U*^23^
Cl1	0.0416 (4)	0.0866 (5)	0.0640 (4)	0.0131 (3)	−0.0136 (3)	0.0047 (3)
N1	0.0337 (8)	0.0409 (9)	0.0366 (8)	0.0104 (6)	0.0033 (6)	0.0145 (7)
N2	0.0386 (8)	0.0400 (9)	0.0372 (8)	0.0141 (7)	−0.0008 (7)	0.0102 (7)
O1	0.0338 (7)	0.0592 (10)	0.0505 (8)	0.0171 (6)	0.0086 (6)	0.0236 (7)
O2	0.0499 (8)	0.0461 (8)	0.0480 (8)	0.0204 (7)	0.0025 (6)	0.0112 (6)
O3	0.0565 (9)	0.0446 (8)	0.0404 (8)	0.0187 (7)	−0.0073 (6)	0.0061 (6)
O4	0.0599 (10)	0.0803 (12)	0.0651 (10)	0.0054 (9)	−0.0098 (8)	0.0438 (9)
O5	0.0434 (8)	0.0775 (12)	0.0759 (11)	0.0141 (8)	0.0111 (8)	0.0468 (10)
C1	0.0322 (9)	0.0411 (10)	0.0360 (9)	0.0112 (7)	0.0069 (7)	0.0149 (8)
C2	0.0407 (10)	0.0479 (11)	0.0419 (10)	0.0191 (9)	0.0087 (8)	0.0134 (9)
C3	0.0351 (10)	0.0637 (14)	0.0478 (11)	0.0208 (9)	0.0065 (8)	0.0211 (10)
C4	0.0336 (9)	0.0597 (13)	0.0421 (10)	0.0090 (9)	0.0007 (8)	0.0147 (9)
C5	0.0411 (10)	0.0482 (12)	0.0403 (10)	0.0110 (9)	0.0017 (8)	0.0074 (9)
C6	0.0364 (9)	0.0451 (11)	0.0369 (9)	0.0133 (8)	0.0060 (7)	0.0147 (8)
C7	0.0421 (10)	0.0462 (11)	0.0419 (10)	0.0190 (9)	0.0021 (8)	0.0097 (9)
C8	0.0335 (9)	0.0433 (11)	0.0386 (10)	0.0139 (8)	0.0034 (7)	0.0134 (8)
C9	0.0335 (9)	0.0395 (10)	0.0351 (9)	0.0113 (7)	0.0076 (7)	0.0154 (8)
C10	0.0345 (9)	0.0444 (11)	0.0402 (10)	0.0099 (8)	0.0049 (7)	0.0151 (8)
C11	0.0904 (19)	0.0536 (15)	0.0500 (13)	0.0273 (14)	−0.0185 (13)	−0.0017 (11)
C12	0.0581 (13)	0.0406 (11)	0.0401 (11)	0.0114 (9)	−0.0023 (9)	0.0059 (9)
C13	0.0763 (17)	0.0797 (18)	0.0606 (15)	0.0248 (14)	0.0260 (13)	0.0355 (14)
C14	0.0481 (13)	0.087 (2)	0.088 (2)	0.0180 (13)	0.0125 (13)	0.0355 (16)
C15	0.0760 (18)	0.098 (2)	0.0528 (14)	0.0339 (16)	−0.0017 (13)	0.0258 (14)
C16	0.093 (2)	0.167 (4)	0.0721 (19)	0.058 (2)	0.0336 (17)	0.075 (2)
C17	0.0537 (14)	0.109 (2)	0.0788 (18)	0.0254 (15)	0.0204 (13)	0.0471 (17)
C18	0.0434 (10)	0.0414 (11)	0.0471 (11)	0.0174 (8)	0.0066 (8)	0.0178 (8)
C19	0.0686 (16)	0.0805 (19)	0.101 (2)	0.0236 (14)	0.0298 (15)	0.0602 (17)

### Geometric parameters (Å, º)

**Table:**

Cl1—C4	1.738 (2)	C6—C7	1.505 (3)
N1—C9	1.272 (2)	C7—C8	1.549 (3)
N1—N2	1.371 (2)	C7—H7A	0.9700
N2—C10	1.350 (3)	C7—H7B	0.9700
N2—H2A	0.86 (3)	C8—C18	1.524 (3)
O1—C8	1.409 (2)	C8—C9	1.537 (3)
O1—H1	0.8200	C11—C12	1.495 (3)
O2—C10	1.206 (2)	C11—H11A	0.9700
O3—C10	1.337 (2)	C11—H11B	0.9700
O3—C11	1.452 (3)	C12—C17	1.362 (3)
O4—C18	1.188 (2)	C12—C13	1.374 (4)
O5—C18	1.308 (3)	C13—C14	1.382 (4)
O5—C19	1.441 (3)	C13—H13	0.9300
C1—C6	1.382 (3)	C14—C15	1.346 (4)
C1—C2	1.390 (2)	C14—H14	0.9300
C1—C9	1.454 (2)	C15—C16	1.347 (4)
C2—C3	1.375 (3)	C15—H15	0.9300
C2—H2	0.9300	C16—C17	1.378 (4)
C3—C4	1.380 (3)	C16—H16	0.9300
C3—H3	0.9300	C17—H17	0.9300
C4—C5	1.381 (3)	C19—H19A	0.9600
C5—C6	1.383 (3)	C19—H19B	0.9600
C5—H5	0.9300	C19—H19C	0.9600
			
C9—N1—N2	117.38 (15)	N1—C9—C8	128.88 (16)
C10—N2—N1	116.91 (15)	C1—C9—C8	108.56 (15)
C10—N2—H2A	122.5 (16)	O2—C10—O3	124.81 (18)
N1—N2—H2A	120.2 (16)	O2—C10—N2	125.41 (17)
C8—O1—H1	109.5	O3—C10—N2	109.77 (16)
C10—O3—C11	113.57 (16)	O3—C11—C12	109.20 (19)
C18—O5—C19	117.33 (18)	O3—C11—H11A	109.8
C6—C1—C2	121.31 (17)	C12—C11—H11A	109.8
C6—C1—C9	109.61 (15)	O3—C11—H11B	109.8
C2—C1—C9	128.97 (17)	C12—C11—H11B	109.8
C3—C2—C1	118.98 (18)	H11A—C11—H11B	108.3
C3—C2—H2	120.5	C17—C12—C13	117.9 (2)
C1—C2—H2	120.5	C17—C12—C11	121.0 (2)
C2—C3—C4	118.93 (17)	C13—C12—C11	121.0 (2)
C2—C3—H3	120.5	C12—C13—C14	120.7 (2)
C4—C3—H3	120.5	C12—C13—H13	119.7
C3—C4—C5	123.02 (18)	C14—C13—H13	119.7
C3—C4—Cl1	118.11 (15)	C15—C14—C13	120.2 (3)
C5—C4—Cl1	118.86 (17)	C15—C14—H14	119.9
C4—C5—C6	117.59 (19)	C13—C14—H14	119.9
C4—C5—H5	121.2	C14—C15—C16	119.8 (2)
C6—C5—H5	121.2	C14—C15—H15	120.1
C1—C6—C5	120.12 (17)	C16—C15—H15	120.1
C1—C6—C7	111.90 (16)	C15—C16—C17	120.6 (3)
C5—C6—C7	127.98 (18)	C15—C16—H16	119.7
C6—C7—C8	104.33 (15)	C17—C16—H16	119.7
C6—C7—H7A	110.9	C12—C17—C16	120.8 (3)
C8—C7—H7A	110.9	C12—C17—H17	119.6
C6—C7—H7B	110.9	C16—C17—H17	119.6
C8—C7—H7B	110.9	O4—C18—O5	125.2 (2)
H7A—C7—H7B	108.9	O4—C18—C8	124.44 (19)
O1—C8—C18	106.74 (14)	O5—C18—C8	110.29 (16)
O1—C8—C9	111.28 (16)	O5—C19—H19A	109.5
C18—C8—C9	107.77 (15)	O5—C19—H19B	109.5
O1—C8—C7	114.79 (16)	H19A—C19—H19B	109.5
C18—C8—C7	111.95 (17)	O5—C19—H19C	109.5
C9—C8—C7	104.18 (14)	H19A—C19—H19C	109.5
N1—C9—C1	122.53 (16)	H19B—C19—H19C	109.5
			
C9—N1—N2—C10	175.94 (17)	C7—C8—C9—N1	173.21 (19)
C6—C1—C2—C3	−2.2 (3)	O1—C8—C9—C1	−132.92 (15)
C9—C1—C2—C3	173.40 (18)	C18—C8—C9—C1	110.37 (17)
C1—C2—C3—C4	0.0 (3)	C7—C8—C9—C1	−8.7 (2)
C2—C3—C4—C5	1.6 (3)	C11—O3—C10—O2	1.6 (3)
C2—C3—C4—Cl1	−177.90 (16)	C11—O3—C10—N2	−179.1 (2)
C3—C4—C5—C6	−1.1 (3)	N1—N2—C10—O2	9.8 (3)
Cl1—C4—C5—C6	178.44 (16)	N1—N2—C10—O3	−169.56 (15)
C2—C1—C6—C5	2.7 (3)	C10—O3—C11—C12	−179.2 (2)
C9—C1—C6—C5	−173.61 (17)	O3—C11—C12—C17	107.2 (3)
C2—C1—C6—C7	−177.73 (18)	O3—C11—C12—C13	−77.1 (3)
C9—C1—C6—C7	5.9 (2)	C17—C12—C13—C14	1.2 (4)
C4—C5—C6—C1	−1.1 (3)	C11—C12—C13—C14	−174.7 (2)
C4—C5—C6—C7	179.5 (2)	C12—C13—C14—C15	0.2 (5)
C1—C6—C7—C8	−11.2 (2)	C13—C14—C15—C16	−2.0 (5)
C5—C6—C7—C8	168.3 (2)	C14—C15—C16—C17	2.4 (6)
C6—C7—C8—O1	133.43 (17)	C13—C12—C17—C16	−0.8 (5)
C6—C7—C8—C18	−104.68 (18)	C11—C12—C17—C16	175.1 (3)
C6—C7—C8—C9	11.5 (2)	C15—C16—C17—C12	−1.0 (6)
N2—N1—C9—C1	−173.57 (15)	C19—O5—C18—O4	0.7 (3)
N2—N1—C9—C8	4.3 (3)	C19—O5—C18—C8	178.73 (19)
C6—C1—C9—N1	−179.68 (17)	O1—C8—C18—O4	−12.3 (3)
C2—C1—C9—N1	4.3 (3)	C9—C8—C18—O4	107.3 (2)
C6—C1—C9—C8	2.1 (2)	C7—C8—C18—O4	−138.7 (2)
C2—C1—C9—C8	−173.92 (19)	O1—C8—C18—O5	169.65 (16)
O1—C8—C9—N1	49.0 (3)	C9—C8—C18—O5	−70.7 (2)
C18—C8—C9—N1	−67.7 (2)	C7—C8—C18—O5	43.3 (2)

### Hydrogen-bond geometry (Å, º)

**Table:**

*D*—H···*A*	*D*—H	H···*A*	*D*···*A*	*D*—H···*A*
O1—H1···O2^i^	0.82	2.13	2.890 (2)	154
N2—H2*A*···O1	0.86 (3)	2.37 (2)	2.891 (2)	119.4 (18)

Symmetry code: (i) −*x*+1, −*y*, −*z*+1.
